# Release of miR-29 Target Laminin C2 Improves Skin Repair

**DOI:** 10.1016/j.ajpath.2023.11.002

**Published:** 2023-11-21

**Authors:** Connor J. Robinson, Lalitha Thiagarajan, Rebecca Maynard, Maneesha Aruketty, Jeremy Herrera, Lewis Dingle, Adam Reid, Jason Wong, Heng Cao, James Dooley, Adrian Liston, Daniela Müllhaupt, Paul Hiebert, Hayley Hiebert, Svitlana Kurinna

**Affiliations:** ∗ Division of Cell Matrix Biology and Regenerative Medicine, Faculty of Biology, Medicine and Health, University of Manchester, Manchester, United Kingdom; † Blond-McIndoe Laboratory, Division of Cell Matrix Biology and Regenerative Medicine, Faculty of Biology, Medicine and Health, University of Manchester, Manchester, United Kingdom; ‡ Division of Pharmacy and Optometry, School of Health Science, Faculty of Biology, Medicine and Health, University of Manchester, Manchester, United Kingdom; § Center for Brain and Disease Research, Flanders Institute for Biotechnology (VIB), Leuven, Belgium; ¶ Department of Microbiology and Immunology, Katholieke Universiteit–University of Leuven, Leuven, Belgium; ‖ Laboratory of Lymphocyte Signaling and Development, The Babraham Institute, Cambridge, United Kingdom; ∗∗ Department of Biology, Institute of Molecular Health Sciences, Eidgenössische Technische Hochschule Zürich (ETH) Zurich, Zurich, Switzerland

## Abstract

miRNAs are small noncoding RNAs that regulate mRNA targets in a cell-specific manner. miR-29 is expressed in murine and human skin, where it may regulate functions in skin repair. Cutaneous wound healing model in miR-29a/b1 gene knockout mice was used to identify miR-29 targets in the wound matrix, where angiogenesis and maturation of provisional granulation tissue was enhanced in response to genetic deletion of miR-29. Consistently, antisense-mediated inhibition of miR-29 promoted angiogenesis *in vitro* by autocrine and paracrine mechanisms. These processes are likely mediated by miR-29 target mRNAs released upon removal of miR-29 to improve cell–matrix adhesion. One of these, laminin (Lam)-c2 (also known as laminin γ2), was strongly up-regulated during skin repair in the wound matrix of knockout mice. Unexpectedly, Lamc2 was deposited in the basal membrane of endothelial cells in blood vessels forming in the granulation tissue of knockout mice. New blood vessels showed punctate interactions between Lamc2 and integrin α_6_ (Itga6) along the length of the proto-vessels, suggesting that greater levels of Lamc2 may contribute to the adhesion of endothelial cells, thus assisting angiogenesis within the wound. These findings may be of translational relevance, as LAMC2 was deposited at the leading edge in human wounds, where it formed a basal membrane for endothelial cells and assisted neovascularization. These results suggest a link between LAMC2, improved angiogenesis, and re-epithelialization.

Worldwide, costs associated with wound care are expected to reach US $15 to 22 billion/year by 2024, demonstrating a significant and rapidly growing clinical need.^1^ In the United Kingdom alone, the treatment of skin wounds costs £8.3 billion per year,^2^ exceeding the cost of managing obesity-related health problems. These projections reflect the increasing clinical burden represented by aberrant wound healing. The normal sequence of events orchestrating an efficient repair process may be disrupted by underlying health conditions such as diabetes or vascular disease, by infections, or by the complexity or the large area of the injury in the case of significant wounds. Wound healing can be typified by three partially overlapping phases: inflammation, new tissue formation, and tissue remodeling.^3^^,^^4^ The molecular events during the early healing stages (inflammation and tissue formation), have been well described through single-cell sequencing and proteomic approaches,^5^ but the mechanisms underlying tissue remodeling are less well characterized.

Recent data implicate miRNAs as important factors in wound repair.^6^, ^7^, ^8^ miRNAs are small noncoding RNAs that target endogenous mRNAs by direct binding to the 3′ untranslated region (UTR). Enhancement of miRNAs with mimics, and inhibition of miRNAs using anti-sense oligonucleotides (ASOs), have both been used to improve wound healing^6^^,^^8^^,^^9^ and have been studied in subsequent clinical trials.^10^^,^^11^ miRNAs 29a, 29b, and 29c have been identified as a miRNA family controlling cell–cell adhesion in the hyperproliferative mouse epidermis.^12^ The murine and human miR-29 family is encoded by two genomic clusters: miR-29a/b1, yielding miR-29a-3p and miR-29b-3p; and miR-29b2/c, yielding miR-29b-3p and miR-29c-3p. All active 3p strands of miR-29a/b/c share the same seed sequence and are thus predicted to bind and share the same mRNA targets.^13^ Their regulatory effects, however, may differ *in vivo* and *in silico*, as mRNAs and miRNAs are expressed in a cell-type–specific manner, and the miR-29 family has additional specific regulatory mechanisms. The miR-29b2/c genomic cluster is silenced by DNA methylation in murine and human keratinocytes,^12^ and miR-29b-3p has a hexanucleotide element, directing it to the nucleus.^14^ Two key transcription factors, p63 (which controls skin development) and nuclear factor erythroid 2–related factor (NRF2) (which activates resistance to oxidants), regulate the expression of miR-29a-3p and miR-29b-3p in human epidermis,^15^ positioning the miR-29 family as a key regulator of skin homeostasis and wound repair.

miR-29a-3p and miR-29b-3p, hereafter referred to as miR-29, regulate collagen production in fibrogenesis^16^ and emerged as a new therapeutic target in a phase 1 clinical trial.^11^ However, crucial information on tissue-specific regulation and on the targets of miR-29 in specific cell types of the wound was largely missing from that study. The present article demonstrates that the genetic deletion of miR-29a/b improves angiogenesis and maturation of the wound granulation tissue, which, in turn, enhances re-epithelialization. miR-29a expression in wounds was detected primarily in the epidermis, with some expression also found in the fibroblasts of the fascia and the granulation tissue. Using published data on wound transcriptomes and predicting miR-29 binding sites in mRNAs with decreased expression during wound healing, major pathways regulated by miR-29 were identified. Among others, pathways including cell adhesion and vascular permeability suggested a role for miR-29 in wound angiogenesis. The loss of miR-29 induced growth of endothelial cells in culture and blood vessels *in vivo*. Moreover, laminin (LAM)-C2 (also known as laminin γ 2 or laminin γ2, a component of laminin 332, or laminin 5) was identified as a target of miR-29, and upon deletion of miR-29 is deposited in the granulation tissue and in the blood vessel lamina. LAMC2 was also identified in blood vessels of human wounds near the leading edge of regenerating epidermis, where it may improve adhesion of epithelial and endothelial cells. The present article describes a novel regulatory function of miR-29 in the wound matrix and suggests that the inhibition of miR-29 and up-regulation of LAMC2 can improve skin repair.

## Materials and Methods

### Animal Experiments

Mice were kept under specific pathogen-free conditions and received water and food *ad libitum*. Mouse maintenance and experiments with mice were approved by the EU/UK veterinary authorities. miR29a/b1 gene KO mice described in the publication by Papadopoulou et al^17^ were backcrossed onto a pure C57BL/6 background. For the analysis of the wound healing process, 8- to 11-week–old mice were anesthetized by isoflurane/oxygen inhalation. An analgesic (buprenorphine, 0.05 to 0.1 mg/kg) was injected s.c. prior to anesthesia, as well as after the initial wound biopsy, as directed by a veterinary surgeon. Two wounding models were used: 6-mm wounds were used for Ki67 staining and quantification of proliferation during healing, and 5-mm wounds were used for morphometry and all of the rest of the staining and quantification. Thus, two anterior and two posterior wounds, or two 6-mm wounds, were generated on both sides of the back lateral to the spine using a 5- or 6-mm hole punch (Stiefel, Brentford, UK), depending on the model. Wounds were collected using an 8-mm biopsy punch post-mortem. Only mice with hair follicles in the telogen phase were wounded and analyzed.

In rare instances, nasal rash developed in female KO mice; these mice were treated in accordance with the animal welfare protocol, which may have introduced variability in the rates of healing and keratinocyte proliferation. As a result, data from only male mice were selected for the final analysis to compare wild-type (WT) and KO wounds, while wounds from female mice were used to test the experimental protocol conditions where necessary.

### Morphometric Analysis

Hematoxylin and eosin–stained, 7-μm paraffin sections of murine excisional wound samples were digitally imaged. Length, area, and cell numbers of wound epithelium, as well as the distance between the wound edges (wound diameter) and the wound gap were analyzed by histomorphometry. Wound closure was calculated as the ratio of the length of the wound epithelium to the wound diameter.

### Human Skin Samples

The use of human chronic wound samples from the Complexwounds@manchester biobank was approved by the UK National Health Service Health Research Authority, with patient consent (protocol number REC#18/NW/0847). The samples used for this study were freshly collected from surgically debrided wounds, selected on the basis that they fit the clinical definition of chronic wounds.^18^ LAMC2, pentachrome, and hematoxylin-eosin staining was performed on serial sections of the same wound to match the details of wound matrix and LAMC2 staining as closely as possible.

### Primary Cell Culture and ASO Transfections

Primary human keratinocytes (PeloBiotech, Planegg, Germany) were maintained in serum-free keratinocyte growth medium (PeloBiotech) at 37°C, 5% CO_2_ on collagen I (Σ) or Coating Matrix Kit Protein (Gibco/Thermo Fisher Scientific, Schwerte, Germany) coated flasks. Cells were used at passages 3 and 4 in all of the experiments stated. Human umbilical cord vein endothelial cells (HUVECs; passage 3) were seeded at 70% to 80% confluence (day 0) and incubated overnight at 37°C, 5% CO2. On day 1, the cells were transfected with miRVana miRNA inhibitors: negative control (catalog number 4464076) and anti–miR-29 (catalog number 4464084-MH12499, -MH10103, and -MH10518; Thermo Fischer Scientific, Waltham, MA; equimolar) at a final concentration of 200 nmol/L, with INTERFERin (catalog number 101000028; Polyplus transfection; Sartorius AG, Göttingen, Germany) according to the manufacturer's protocol. The cells were re-transfected on day 3 and collected for the RNA analysis or functional assays on day 4. All transfections were performed in biological and technical triplicates.

### Cell Viability Assay

The PrestoBlue cell viability assay (catalog number P50200, Invitrogen/Thermo Fisher Scientific, Horsham, UK) was performed on day 1 prior to transfection, and on day 4 before the collection of the cells for RNA extraction or functional assay, according to manufacturer's protocol. The results were then normalized to the corresponding day-1 readings to generate fold-change values. All transfections were performed in biological and technical triplicates.

### Tube Formation Assay

HUVECs (30,000 cells/cm^2^) were seeded in Matrigel-coated (catalog number 11573620; Corning Life Sciences, Corning, NY) 96-well angiogenesis μ-plate (catalog number 89646; Ibidi, Gräfelfing, Germany) and incubated for 24 hours in growth medium (catalog number C-22110; PromoCell, Heidelberg, Germany) at 37°C, 5% CO_2_. For better visualization of the cells, the cells were stained with Calcein AM (BioLegend, Fell, Germany) according to the manufacturer's protocol. The cells were imaged using an IncuCyte live-cell analysis system (Sartorius) in the Essen BioScience Angiogenesis Analysis module was used to identify and measure tube length and branch point formation.

### miRNA *in Situ* Hybridization Combined with Immunofluorescence

Based on previous work on miRNA *in situ* hybridization analysis with fluorescently labeled locked nucleic acid probes^17^ [fluorescence *in situ* hybridization (FISH)], a modified miR-29 *in situ* hybridization protocol was developed in which the probes specifically hybridizing miR-29a, miR-29b, or a scramble sequence of the same length were modified at the backbone and labeled with Cy2. Briefly, cryopreserved mouse wound biopsy samples were sectioned (14 μm) and fixed with 4% paraformaldehyde. miR-29a and scrambled sequences (*n* = 48), synthesized and Cy2-labeled by RiboTask (Langeskov, Denmark), were hybridized at 63°C (miR-29a) or 55°C (scrambled). Sections of the mouse hippocampus, which expresses high levels of miR-29a, were used as a positive control, whereas skin sections from miR-29a/b1 gene KO mice (*n* = 4) hybridized with the miR-29a probe, as well as sections hybridized with the scrambled probe, served as negative controls to find the optimal temperature for hybridization and washing steps. The antibody for keratin K14 (BioLegend) were added at the last step of the wound section incubation and visualized using the secondary anti-rabbit Cy3-labeled antibody.

### RT-qPCR and miRNA Expression Analysis

Total RNA from mouse tissues was isolated by homogenization of the entire wound with the Ultra-Turrax rotating blade (IKA, Staufen, Germany) in excess TRIzol reagent (Invitrogen/Thermo Fisher Scientific) and purified following the manufacturer's protocol. Total RNA from the cultured cells was isolated using TRIzol, and the cDNA synthesis for mRNAs was performed with random hexamer primers using the SensiFast cDNA synthesis kit (Bioline, London, UK) and the manufacturer's protocol. cDNA levels were measured by quantitative real-time RT-PCR (RT-qPCR) using *ACTB* as reference, and primers listed in the corresponding Table 1 miRNA cDNA were synthesized using the MultiScribe Reverse Transcription kit (Invitrogen/Thermo Fisher Scientific) following the manufacturer's protocol. The TaqMan miR-specific assays (Ambion/Thermo Fisher Scientific) were used to measure miR-29 levels, normalized to RNU48.

**Table 1:** Primers and Oligonucleotide Sequences

Gene name	Primer/probe number or sequence
RNA primers for RT-qPCR	
*ACTB*	Assay ID Hs99999903_m1
*LAMC2*	Assay ID Hs01043717_m1
sRNA primers/assays	
RNU	TaqMan assay ID: 001006
miR-29a/b/c-3p	TaqMan assay IDs: 002112, 000413, 000587, corr.
miRNA ASOs	
ASO miR-29a/b/c-3p	5′-G**Cy3**AUUUCAGAUGGUGCU-3′
ASO scramble	5′-G**Cy3**GCCGUUAUAUUAUGG-3′
mirVana miRNA negative control 1	Catalog no. 4464058
mirVana hsa-miR-29a-3p	Catalog no. 4464066; assay ID MC12499
mirVana hsa-miR-29b-3p	Catalog no. 4464066; assay ID MC10103

IDs/catalog numbers from Thermo Fisher Scientific. TaqMan assay IDs for the mRNA expression analysis are listed, followed by the TaqMan assay IDs for sRNA probes. In the miR-29–specific and scramble anti-sense oligonucleotides (ASOs), the Cy3 label position is shown in bold, indicating that it is not a part of the RNA sequence but covalently attached to the first G nucleotide from the 5′ end. miR-29 or negative (nonspecific) control mirVana mimics are listed with the corresponding assay IDs for miR-29a and miR-29b. The miR-29c mimic was not used as it differs by only 1 nucleotide outside of the seed sequence match and is otherwise identical to miR-29a in structure and function.

### Luciferase Assays

Ten thousand HEK293T cells were seeded in Dulbecco's modified Eagle's medium (Life Technologies, Carlsbad, CA) and 10% fetal bovine serum (Gibco) in 24-well plates. The cells were transfected with 20 ng of dual luciferase reporter plasmid (Promega, Southampton, UK) with the 3′UTRs for the *LAMC2* WT or mutant sequences cloned behind the firefly luciferase gene. Relative luciferase activity was measured after transfection with miR-29 or nonspecific oligonucleotides on a GLOMaX Luminometer (Promega) using Renilla luminescence (Promega) as a control to normalize the level of transfection. The error bars in the images represent the SDs of at least three independent transfections. The Q5 site-directed mutagenesis kit (New England BioLabs, Hitchin, UK) was used to mutate 3′UTR of *LAMC2* at the site of minimal 6 nucleotides required for miR-29 (seed sequence, UGGUGC) and compared to the levels of the WT 3′UTR inhibition by miR-29 or nonspecific oligonucleotides (negative control 1) (Table 1).

### Immunostaining

Sections of freshly frozen mouse wounds were fixed with 4% paraformaldehyde and stained with fluorescein isothiocyanate–conjugated anti-Lamc2 (clone D4B5; EMD Millipore, Temecula, CA), anti–integrin α_6_ (Itga6; catalog number MCA699GA; Bio-Rad, Watford, UK) and DAPI. Deconvolution of confocal images was performed using LAS X software (Leica Microsystems, Wetzlar, Germany). For analysis of wound bed and vasculature, the sections were stained with anti-vimentin (catalog number EPR3776; Abcam, Cambridge, UK), anti–phospho-vascular endothelial growth factor receptor (VEGFR)-2 (clone 19A10; Cell Signaling Technology, Danvers, MA), α-smooth muscle actin (SMA; clone 1A4, R&D systems), and anti-CD31 [platelet endothelial cell adhesion molecule (PECAM)-1, clone D8V9E; Cell Signaling Technology, Danvers, MA], followed by the Cy2-, Cy3-, or Cy5-labeled secondary antibody. Sections from paraffin-embedded human complex skin wounds were stained with anti-LAMC2/laminin γ2 antibody (mouse monoclonal, clone D4B5; Merck Millipore, now MilliporeSigma, Burlington, MA) and counterstained with hematoxylin.

miR-29 targets were predicted using a list of mRNA transcripts from published RNA sequencing, which was done using a similar dorsal wound model in mice of the same genetic background as the WT littermates in the present study.^19^ Predicted target transcripts of miR-29 were obtained from the micro-TDS tool in DIANA (*http://diana.imis.athena-innovation.gr/DianaTools*) and TargetScan software version 8 (*https://www.targetscan.org/vert_80*). Predicted miR-29 target transcripts with a *z*-score of >0.3 were used for further comparison with the RNAs that significantly changed expression in mouse back wounds compared to unwounded skin.^19^

### Data Sets and Statistical Analysis

Data from three mice per group were used in each analysis using one- or two-way analysis of variance for all data applicable, unless otherwise mentioned. All experiments were done in technical and biological triplicates.

## Results

### miR-29a Is a Major Functional miRNA in Wound Epidermis

The aim of this study was to identify new mechanisms that could potentially be exploited to improve cutaneous wound healing. miRNAs have short targetable sequences, and miRNA mimics and antagonists are already being tested in relevant clinical trials.^10^ The focus was on miR-29a/b1 because of the conserved function of this cluster in murine and human epidermis.^12^^,^^15^ Earlier findings established a new mechanism whereby the expression of the miR-29a/b1 cluster was activated by joint activity of NRF2 and p63, resulting in the increase in miR-29a in the suprabasal (but not the basal) keratinocytes involved in the growth and repair of epidermis.^15^

**Figure 1:**
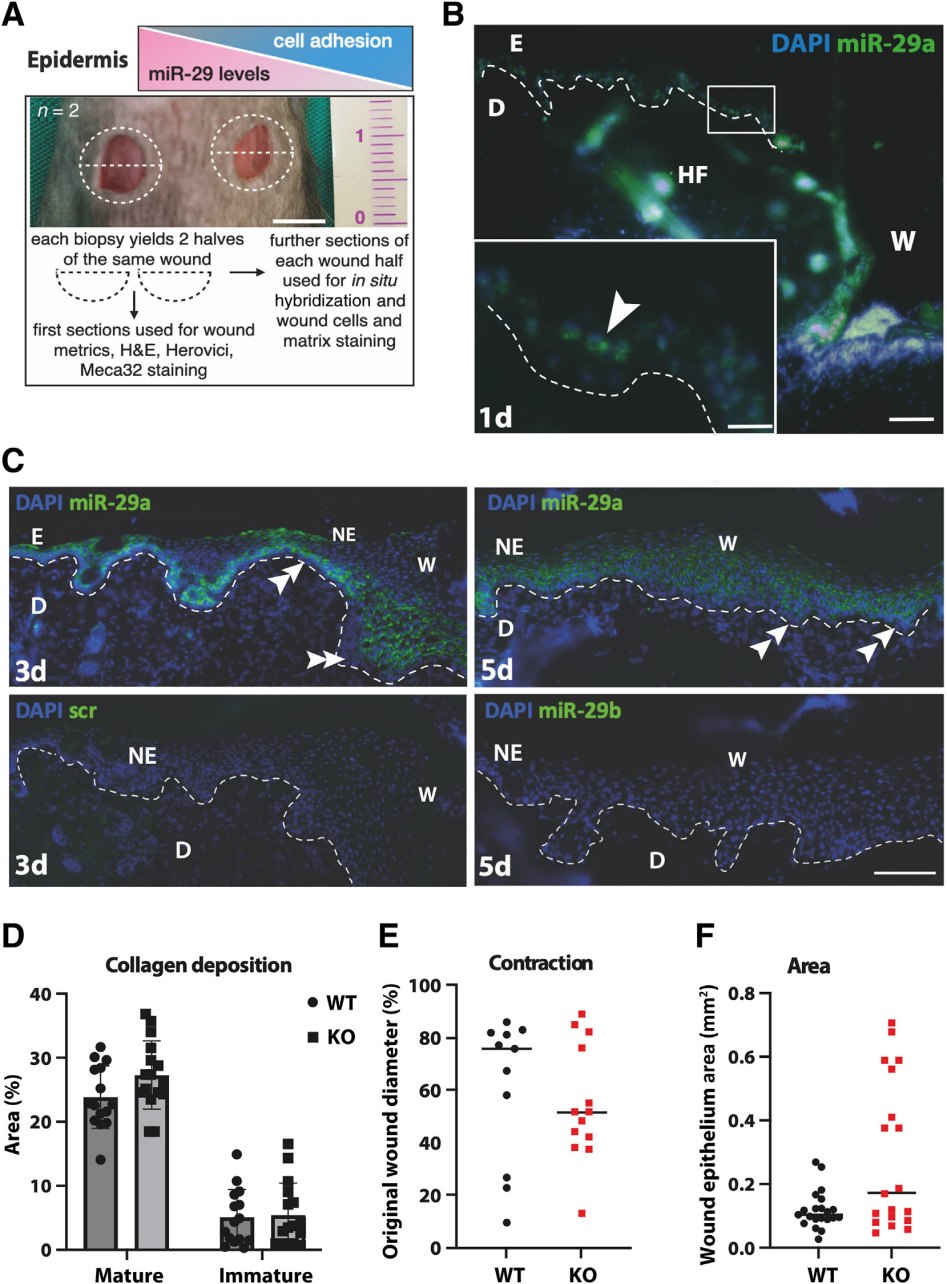
miR-29a is expressed in suprabasal neoepidermis and in the granulation tissue of healing wounds in mice. **A:** The role of miR-29 in cell adhesion in the epidermis was tested using the skin wound model. Two wounds (**dashed lines**) are shown in the mouse dorsum at day 0 after biopsy (middle), followed by the experimental plan (bottom). A decrease in miR-29 may affect cutaneous repair through the up-regulation of mRNA targets of miR-29. **B** and **C:** miR-29a in sections of wounds harvested at days 1, 3, and 5 after biopsy [fluorescence *in situ* hybridization (FISH); nuclei counterstained with DAPI]. The **inset** corresponds to the **boxed region** at greater magnification. **Dashed lines** demarcate the border between the dermis and the epidermis. **Arrowhead** shows suprabasal keratinocytes positive for miR-29a at the edge of the wound; **double arrowheads** point to the basal layer of regenerating epidermis void of miR-29. Scrambled probe was used to control the background and is shown at day 3 for representation; the image at day 5 is representative of the undetected expression of miR-29b. **D:** Quantification of positively stained pixels in day-5 wound sections from WT and KO mice; representative images are shown in Figure 2H. **E:** Wound contraction, assessed as the percentage of the original wound diameter, in WT and KO wounds, as shown in Figure 2A. **F:** Area of newly formed wound epidermis, as shown in Figure 2A. Data are expressed as actual values and means ± SD. *n* = 12 wounds per genotype. Scale bars: 5 mm (**A**); 10 μm (**B,** inset); 50 μm (**B** and **C**). E, epidermis; D, dermis; H&E, hematoxylin and eosin; HF, hair follicle; NE, neoepidermis; W, wound bed.

**Supplemental Figure S1:**
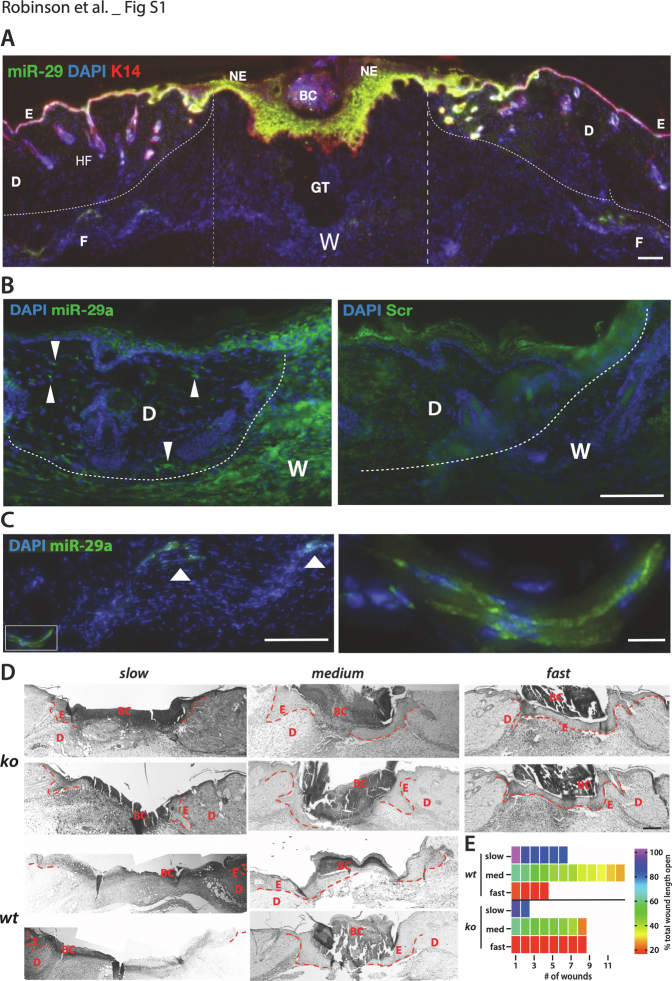
Loss of miR-29 improves wound closure but not proliferation or wound contraction. **A:** miR-29a and keratin K14 in a wound at day 5 of healing FISH combined with immunofluorescence]. Nuclei were counterstained with DAPI. **Dotted lines** demarcate the border of the wound granulation tissue and the contracted dermis; **dashed lines** show the approximate wound (W) edges made by the biopsy punch before contraction. **B:** miR-29a expression in dermal cells (**arrowheads**) and in the granulation tissue of the wound bed (FISH and scrambled probe). **Dotted lines** demarcate the border of the wound granulation tissue and the contracted dermis (D). **C:** Expression of miR-29a in fibroblasts of the fascia on the border of the wound granulation tissue (**arrowheads**) (FISH). Elongated facia fibroblasts specifically stain with miR-29a (**boxed region**, magnified on the right). **D** and **E:** Representative images (**D**) and a histogram (**E**) of wound closure, showing the differences between the numbers (%) of total wound closed, in KO and WT mice. **Dashed lines** demarcate the length of the wound epidermis (E) as the wound closes. Scale bars: 100 μm (**A–C**, left); 10 μm (**C**, right); 200 μm (**D**). BC, blood clot; E, epidermis; F, fascia; GT, granulation tissue; HF, hair follicle; NE, neoepidermis.

To test miR-29 function in regenerating skin, a mouse cutaneous dorsal wound closure model was used for accessing the expression of miR-29 and to measure the growth of the epidermis in the absence of miR-29 (Figure 1A). Indeed, the *in situ* hybridization using fluorescently labeled probes for miR-29 was used to detect miR-29a in the suprabasal epidermis of regenerating wound at days 1, 3, and 5 (Figure 1, B and C), and in the wound matrix forming beneath it from the provisional granulation tissue (Supplemental Figure S1, A and B). miR-29a was also expressed in dermal fibroblasts adjacent to the wound, also known as fascia (Supplemental Figure S1C). miR-29 was not expressed in keratin 14–positive basal keratinocytes of the wound, which was consistent with previous findings.^15^ Mature miR-29a was detected in the suprabasal keratinocytes of regenerating epidermis at days 3 and 5 after wounding, while miR-29b was below the level of detection at all time points tested (Figure 1C). It was hypothesized that the low levels of miR-29 allow for basal cell adhesion and proliferation, whereas an increase in miR-29 expression in suprabasal epidermis fine-tunes cell-to-cell adhesion, keeping it flexible and dynamic.^12^ This hypothesis is illustrated by a simplified diagram (Figure 1A), suggesting an important function of miR-29 in cutaneous wound healing. Thus, miR-29a, hereafter also referred to as miR-29, is the most functional miR-29 family member in normal and regenerating skin, with a potential to suppress cell adhesion during wound repair.

### miR-29 Regulates Re-Epithelialization, Formation of Wound Matrix, and Angiogenesis

**Figure 2:**
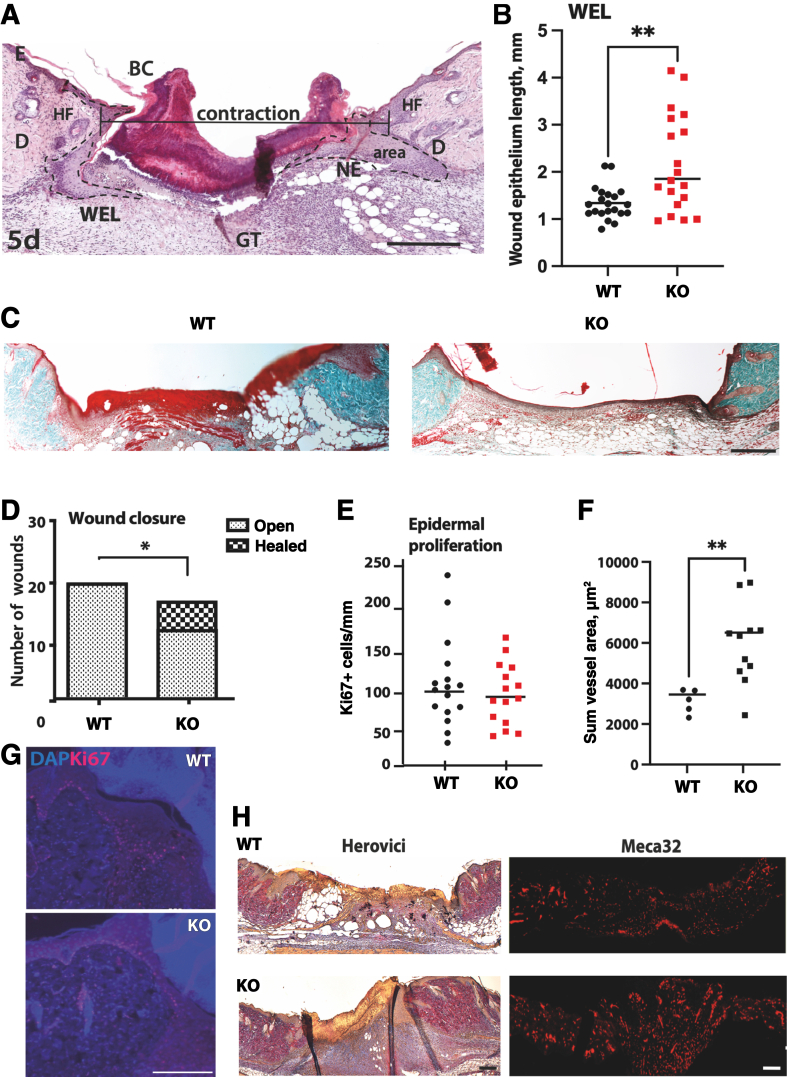
Loss of miR29a promotes wound re-epithelialization, closure, and deposition of Lamc2. **A** and **B:** Representative image (**A**) of morphometrics of wound epithelium length [WEL, assessed as length between wound edges (**B**), marked by hair follicles on both sides of the wound], area, and distance from dermal compartments on each side of the wound at 5 days. **Dashed lines** demarcate the border of the neoepidermis (NE). **C** and **D:** Representative images (**C**, Trichrome staining) and quantification (**D**) of open and closed wounds in WT and KO mice at day 5. **E** and **G:** Representative images (**G**) and quantification (**E**) of Ki67^+^ cells in the wound epidermis of WT and KO littermates at 5 days after biopsy. **F** and **H:** Distribution of mature (purple) and immature (blue) matrix fibers [**H** (left; Herovici staining for collagens I and III), quantified in Figure 1D] in the wound bed in WT and KO mice at day 5, with a difference in distribution of endothelial cell marker Meca32 (**H**, right), quantified as the sum of the areas of staining (**F**). Data are expressed as actual values (**D**) and as actual values and means (horizontal lines) (**B**, **E**, and **F**). *n* = 5 to 11 wounds per genotype (**F**). ∗*P* < 0.05 (χ^2^ test), ∗∗*P* < 0.01 (*U*-test). Scale bars: 500 μm (**A** and **C**); 50 μm (**G**); 200 μm (**H**). E, epidermis; BC, blood clot; D, dermis; GT, granulation tissue; HF, hair follicle; W, wound bed.

**Supplemental Figure S2:**
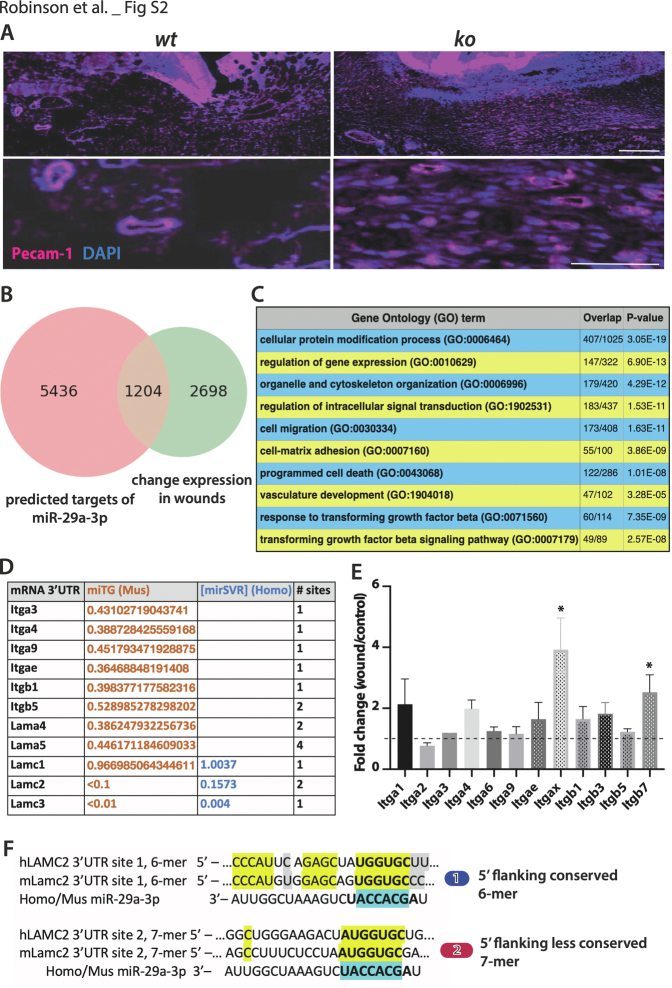
Potential direct targets of miR-29 with a function in cell–cell adhesion. **A:** Mouse wound sections at day 5 after biopsy, stained with the endothelial cell marker Pecam1. **B:** Venn diagram shows the numbers of predicted targets of miR-29 change expression in dorsal wounds of WT mice compared to unwounded skin. **C:** Gene Ontology (GO) analysis of predicted miR-29 targets up-regulated in wounds suggests a role for miR-29 in suppressing regulators of protein modifications, gene expression, cell signaling, migration, and adhesion (*https://geneontology.org*). GO terms are sorted based on the lowest *P* value and highest overlap between predicted miR-29 targets and the number of mRNAs within the category. **D:** miR-29 binding scores for selected targets involved in cell adhesion. For mouse 3′UTRs, the miTG score from the DIANA tool and for humans was used, and the miR-SVR score from the Miranda algorithm (converted to positive value for easier comparison) was used. **E:** RNA levels of wound-induced integrin, as determined by RNA sequencing [fragments per kilobase of transcript per million mapped reads (FPKM), fold change compared to control (unwounded) skin, **dashed line**]. Plotted are results from skin/wound isolations. **F:** Analysis by TargetScan software version 8 (*https://www.targetscan.org/vert_80*) indicated two putative miR-29s–binding sites in the human *LAMC2* 3′UTR (positions 611 to 627 and 1430 to 1436), with the 100% conservation to the two mouse *Lamc2* 3′UTR sites identified in the present study and highlighted in yellow and in bold. Note the high degree of homology in the flanking regions of the first site comprising a 6-mer miR-29 seed match, highlighted in yellow, and similar (pyrimidine) nucleotides in the flanking regions, highlighted in gray. The second site shows less homology in the flanking regions between the murine and human *LAMC2* mRNA; however, it had a 100% match in the 7-mer seed site. Data are expressed as means ± SD. *n* = 4 mice. Scale bars: 200 μm (**A**, top); 50 μm (**A**, bottom).

Enhancing cell adhesion during tissue repair in response to a decrease in miR-29 could be beneficial. To test this hypothesis, the miR-29a/b1 cluster KO mouse model^17^ was used for studying the healing of full-thickness wounds in the absence of miR-29^20^ and wounds at 3, 5, and 7 days were compared in KO mice and their WT littermates. Importantly, with the deletion of miR-29, collagen deposition and wound contraction, significant contributors to skin repair in mice, were not excessive (Figure 1, D and E), and re-epithelialization area also was not increased (Figure 1F). The total wound epidermal length, the area of newly regenerated epidermis, and wound contraction were measured as shown in Figure 2A. Interestingly, wound epidermal length was significantly greater in KO mice compared to the WT littermates (Figure 2B). The number of completely closed wounds also demonstrated a clear advantage in cutaneous healing in KO over the WT mice (Figure 2, C and D). The total wound epidermal length in KO mice showed a shift toward improved healing, leaving open wounds at about 20%, while WT wounds had a more gradual distribution of healing rate (Supplemental Figure S1, D and E). These results suggest that faster re-epithelialization in the absence of miR-29 depends on keratinocyte proliferation and migration rather than on deposition of the extracellular matrix by fibroblasts. However, the proliferation of keratinocytes was not changed in response to the loss of miR-29 (Figure 2, E and G). Because of this unexpected finding, the wound bed was examined in more detail. The wound repair process is highly dependent on the formation of the provisional matrix of the granulation tissue, where miR-29a expression was also detected (Supplemental Figure S1B). The granulation tissue under the regenerating epidermis was therefore examined. A marker of angiogenesis, Meca32, revealed a greater density of blood vessels in the wound matrix of KO mice (Figure 2, F and H). The wound gap in the KO mice was filled with apparently more structured granulation tissue (Figure 2H), which includes many small and newly forming blood vessels. Pecam-1, a marker of endothelial cell adhesion, confirmed this observation (Supplemental Figure S2A), suggesting that miR-29 regulates angiogenesis in the wound matrix.

To identify the molecular mechanism behind the observed changes in wound healing in miR-29 KO mice, the mRNAs that significantly changed expression during the course of wound healing in WT mice were extracted from published RNA-seq^19^ and compared to known or predicted targets of miR-29. Functional analysis of mRNAs that were up-regulated during wound healing and that had confirmed or predicted binding of miR-29a in their 3′UTRs were collated into Supplemental Table S1 and Supplemental Figure S2B. The top 10 significantly enriched Gene Ontology categories included regulators of wound repair that function as regulators of protein modifications, gene expression, signal transduction, cell migration, and cell-to-matrix adhesion (Supplemental Figure S2C). A significant percentage of mRNAs potentially targeted by miR-29 in wounds also revealed vasculature development (Supplemental Figure S2C). Several of these mRNAs, such as those encoding VEGFA and AKT3, are known miR-29 targets,^21^^,^^22^ but the majority represent yet-unknown downstream effectors of miR-29 and strongly warrant further investigation. Interestingly, in one study, overexpression of miR-29b (which can potentially bind the same mRNAs as miR-29a) inhibited vascularization of the wound bed in a dorsal cutaneous wound model through an undefined mechanism.^23^ The findings from that and a more recent study describing improved healing of large-area diabetic mouse wounds^24^ confirm the present findings—the deletion of the miR-29a/b1 cluster promoted angiogenesis during skin repair.

**Figure 3:**
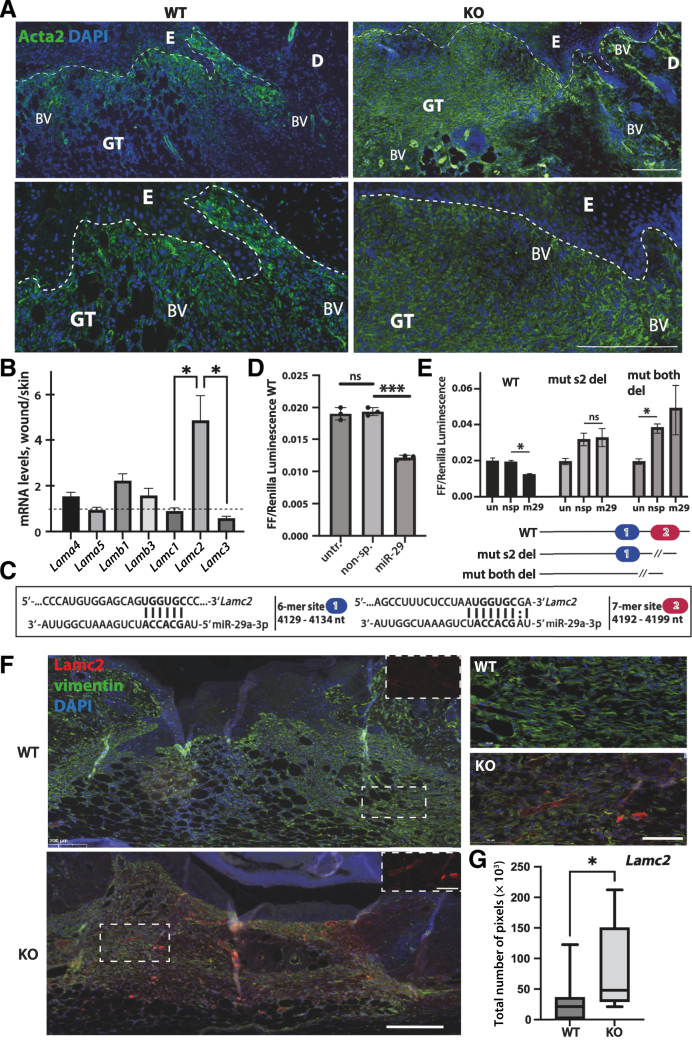
Loss of miR-29a promotes changes in provisional wound matrix. **A:** α-Smooth muscle actin (Acta2) staining of mouse wounds shows faster maturation of provisional matrix in miR-29 KO wounds compared to WT. **Dashed lines** mark the border between the epidermis (E) and granulation tissue (GT) of the wound. **B:** Laminin mRNA levels, as determined by RNA sequencing [fragments per kilobase of transcript per million mapped reads (FPKM), fold change compared to control (unwounded) skin, **dotted line**]. **C:** Two positions and mRNA sequences of the binding sites matching the miR-29 seed sequence (**vertical lines**; **dotted vertical line** shows enhanced interaction between purine bases without a Watson-Crick match) within the 3′ untranslated region (UTR) of the *Lamc2* mRNA. **D:** Luciferase assays show inhibition of the mRNA of the fused firefly (FF)-WT-*Lamc2-*3′UTR reporter gene by miR-29 compared to a nonspecific (non-sp.) oligonucleotide treatment. Renilla (Promega) gene expressed from the same plasmid was used as a normalization control for transfections. **E:** Nested analysis of the WT and mutant FF-fused 3′UTR of *Lamc2* with site 2 or both sites deleted (bottom). **F** and **G:** Lamc2 staining in wounds from WT and KO mice, counterstained with vimentin to mark the wound granulation tissue. **Boxed regions** allow a closer look at the Lamc2 deposition in wound granulation tissue and demonstrate how areas were chosen for the quantification analysis (**G**). Data are expressed as means ± SD (**B** and **E**); as actual values and means ± SD (**D**); and as minimums, maximums, and medians (interquartile ranges) (**G**). *n* = 4 mice (**A**). ∗*P* < 0.05 [**A** and **E**, one-way analysis of variance (ANOVA); **G**, *U*-test] and ∗∗∗*P* < 0.001 (one-way ANOVA). Scale bars: 200 μm (**A** and **F**, left); 50 μm (**F**, right). BV, blood vessel; D, dermis; ns, not significant; nsp, nonspecific; nt, nucleotides; un, untreated; untr, untreated.

The production of provisional wound matrix in the granulation tissue is regulated by the differentiation of fibroblasts into myofibroblasts,^25^ with α-smooth muscle actin (ACTA2), a protein expressed by myofibroblasts. Interestingly, miR-29 KO mice had visibly more Acta2 in their wound granulation tissue where it distributed in a more dispersed manner, possibly also involving myofibroblasts within the blood vessel wall (Figure 3A). Myofibroblasts within the wound actively secrete collagen I A1 and III A1 (Col1a1 and Col3a1); while these are both confirmed miR-29 targets *in vitro*, neither was directly suppressed by miR-29 in mouse skin.^26^ ACTA2, however, is not a direct target of miR-29 but can increase in response to the activation of the transforming growth factor β pathway (Supplemental Figure S2C and Putra et al^27^). These results suggest that miR-29 regulates wound matrix maturation by previously undescribed targets and pathways.

### Loss of miR-29 Results in High LAMC2 Expression in Regenerating Wound and Reveals a Role for miR-29 as a Regulator of Endothelial Cells

Given that the cell–cell adhesion is the sole miR-29–regulated process confirmed in mouse skin *in vivo*,^12^ the regulators of cell–matrix adhesion, integrins and laminins, were tested for possible suppression by miR-29. LAM332 (consisting of LAMA3, LAMB3, and LAMC2) is present in the epidermal/dermal basal membrane (BM), where it interacts with the integrins of basal keratinocytes.^28^ Among the integrins with a miR-29 seed sequence match in the mRNA 3′UTR, the expression of only *Itgax* and Itgb7 were significantly changed in the mouse dorsal wounds compared to unwounded skin (Supplemental Figure S2, D and E). Among laminins, only *LAMC2* had two sites complementary to the seed of miR-29, conserved in both mice and humans (Supplemental Figure S2, D and F), and was significantly up-regulated in wounds (Figure 3, B and C). While human LAMC2 had been previously confirmed as a target of miR-29 on both sites in the 3′UTR,^13^ no reports demonstrated mouse Lamc2 as a direct target of miR-29. Both miR-29 binding sites of mouse Lamc2 may contribute to the inhibition of the mRNA, with the 7-mer binding miR-29 more strongly compared to the 6-mer site.^29^ Indeed, when cloned into the 3′UTR of the firefly reporter gene, the WT *Lamc2* 3′UTR was significantly suppressed by miR-29 (Figure 3D), and this suppression was reversed when the 7-mer site was deleted (Figure 3E). A significant increase in luciferase activity of the firefly mutant 3′UTR with both miR-29 seed sites deleted (Figure 3E) may be attributable to the release of inhibition of the firefly mRNA by endogenous levels of miR-29 in HEK293T cells. Taken together, these results demonstrate that miR-29 binding to the 3′UTR of *Lamc2* mRNA repressed mRNA to protein translation, resulting in lower levels of functional protein. This effect was reversed upon deletion of the UGGUGC sequence of the seed of miR-29 (Figure 3, C–E).

**Supplemental Figure S3:**
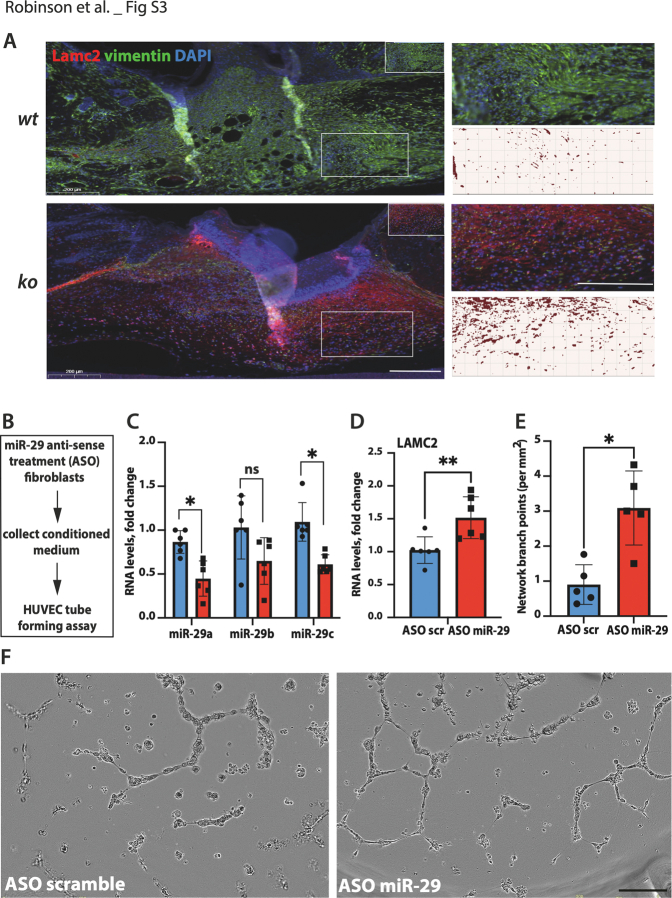
Inhibition of miR-29 promotes laminin deposition in wound matrix and endothelial cell tube formation in a paracrine manner. **A:** Lamc2 staining in wounds from WT and KO mice, counterstained with vimentin. **Boxed regions** allow a closer look at the areas of Lamc2 deposition and demonstrate how the red signal for Lamc2 was isolated and quantified in Figure 3E. **B:** Experimental setup of HUVEC tube assay in fibroblast-conditioned medium. **C** and **D:** Levels of miR-29 inhibition (**C**) and LAMC2 up-regulation (**D**) in fibroblasts after miR-29 anti-sense oligonucleotide (ASO) treatment as compared to scramble ASO. **E** and **F:** HUVEC network branching quantified using IncuCyte (Sartorius) (**E**) and representative images (**F**). miR-29a and *LAMC2* levels were determined after transfection by RT-qPCR. All experiments were performed in triplicate. Data are expressed as means ± SD. ∗*P* < 0.05 and ∗∗*P* < 0.01 (**C** and **D**, *U*-test; **E**, unpaired *t*-test). Scale bars: 200 μm (**A**); 100 μm (**F**). ns, not significant versus control.

If Lamc2 is repressed by miR-29, a release of Lamc2 expression in miR-29 KO wounds could at least partially explain their improved re-epithelialization. The Lamc2 protein levels in WT and KO mouse wounds were checked with immunostaining. Unexpectedly, Lamc2 expression was increased away from the epidermis and deep into wound granulation tissue (Figure 3, F and G, and Supplemental Figure S3A). In most KO wounds, the expression was so prominent that it blocked the signal for vimentin, an intermediate filament found in mesenchymal cells and a general marker of wound matrix (Figure 3F and Supplemental Figure S3A). When quantified, Lamc2 expression showed variability in KO wounds but, overall, was significantly greater in the wound granulation tissue of KO mice (Figure 3G).

**Figure 4:**
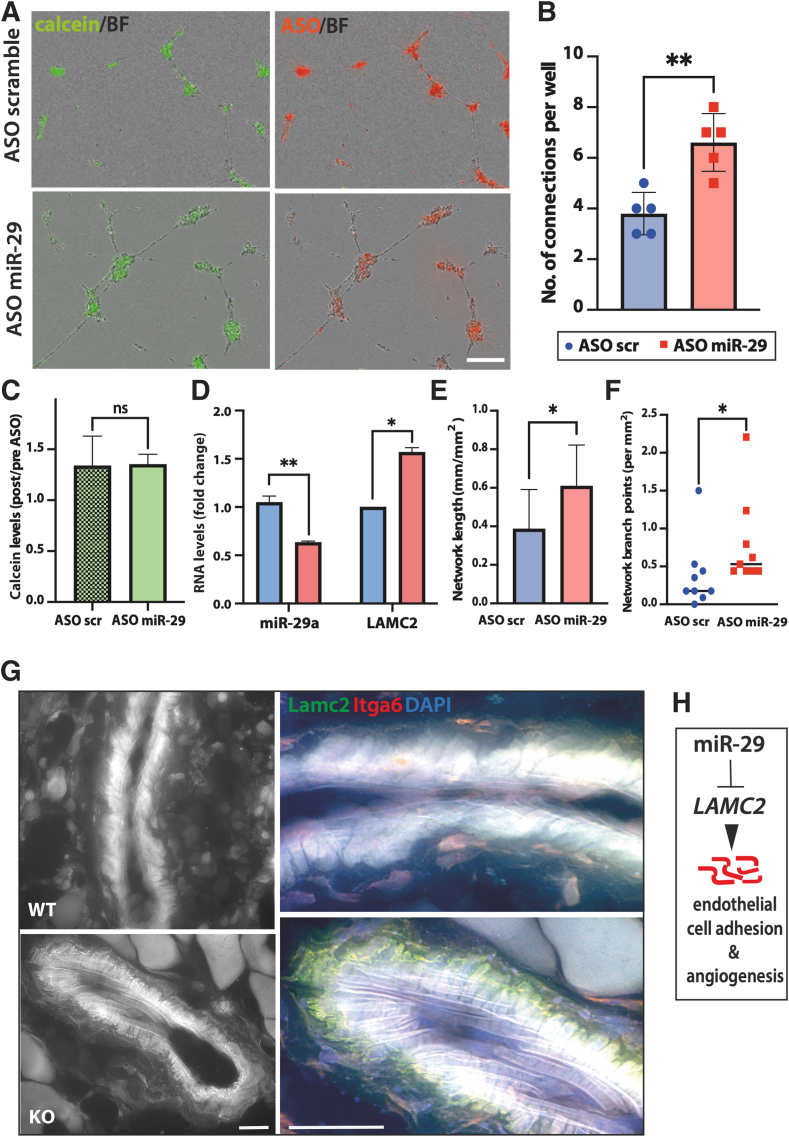
Loss of miR-29 promotes endothelial cell tube formation, rescues LAMC2 expression, and enhances Lamc2 deposition in blood vessels of mouse wounds. **A:** HUVECs were transfected twice with Cy3-labeled anti–miR-29 oligonucleotide (ASO) or nonspecific control (scrambled) oligonucleotide with a day of recovery in between the transfections. **B**, **E**, and **F:** Tube formation was estimated by seeding transfected cells on Matrigel, staining with Calcein AM (BioLegend), and imaging using IncuCyte (Sartorius) to detect the network connectivity (**B**), length (**E**), and branching (**F**). **C:** Viability was assessed using PrestoBlue reagent (Invitrogen/Thermo Fisher Scientific) before and after the two transfections. miR-29 ASO had no significant (ns) effect on viability. **D:** miR-29a and *LAMC2* levels were determined after transfection by RT-qPCR. **G:** Lamc2 and its predicted ligand, integrin α_6_ (Itga6), show co-localization in the blood vessel basal membrane in wounds from KO but not WT mice (high-resolution deconvoluted immunofluorescence staining). **H:** miR-29 inhibits regulators of endothelial cell adhesion, such as *LAMC2*, and regulates angiogenesis. Data are expressed as actual values and means (SD) (**B**), as means ± SD (**C–E**), and as means (**F**). ∗*P* < 0.05 and ∗∗*P* < 0.01 (**B**, **E**, **F**, unpaired *t*-test; **D**, *U*-test). Scale bars: 200 μm (**A**); 20 μm (**G**). BF, brightfield.

These results suggest a role for miR-29 as a regulator of adhesion of endothelial cells. HUVECs were used to test this concept. When plated into the three-dimensional matrix, HUVECs can form long adhesive protrusions and even a simple network of tubes, partially reflecting the processes in endothelial cell growth and BM attachment during angiogenesis. With partial inhibition of all miR-29s using fluorescently labeled ASOs, the number of connections between individual cells, the extent of branching, and the network formed by HUVECs were significantly improved, while *LAMC2* mRNA was also significantly up-regulated (Figure 4, A–F). Interestingly, similar results could be achieved by the interaction between fibroblasts and endothelial cells in a paracrine manner by transfecting fibroblasts with miR-29 ASO and adding the medium conditioned by the fibroblasts onto nontransfected HUVECs (Supplemental Figure S3, B–F). Fibroblast-derived factors, released by the inhibition of miR-29, increased network branching in HUVECs even further than the endogenous inhibition of miR-29 within these cells (Supplemental Figure S3, E and F). These results are in line with previously reported regulation of tumor necrosis factor α–induced adhesion in human aortic endothelial cells and human umbilical vein smooth muscle cells, which was significantly decreased after transfection with miR-29a and rescued by a miR-29a inhibitor.^30^ While many other mRNA targets of miR-29 could be involved in improved tube formation *in vitro* and angiogenesis *in vivo*, the present results suggest a previously unknown role for LAMC2 in the interaction of endothelial cells with the matrix.

### LAMC2 Is Deposited in Blood Vessels in miR-29a KO Mouse and Human Wounds

**Supplemental Figure S4:**
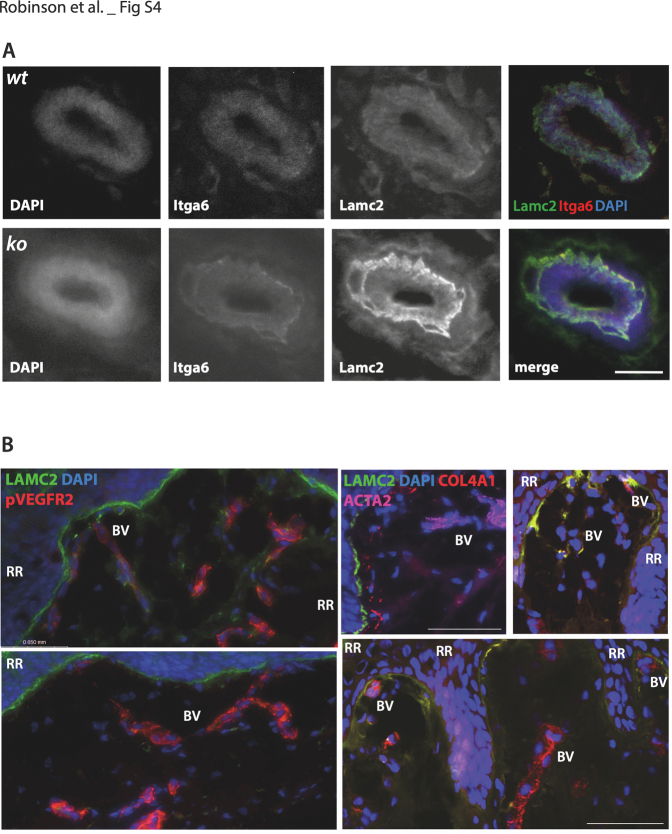
LAMC2 deposition in blood vessels of wounds from murine and human skin. **A:** Lamc2 staining in wounds from WT and KO mice, counterstained with integrin α_6_ (Itga6). **B:** Human skin sections stained with LAMC2, phospho–vascular endothelial growth factor receptor (p-VEGFR)-2, and α-smooth muscle actin (ACTA2) (nuclei counterstained with DAPI). BV, blood vessels; RR, rete ridges. Scale bars: 20 μm (**A**); 50 μm (**B**).

To confirm that this interaction occurs in wounds *in vivo*, wound granulation tissue was visualized at a higher resolution and Lamc2 was detected at the BM of blood vessels (Figure 4G and Supplemental Figure S4A). After deconvolution, the signal for Lamc2 co-localized with Itga6 on endothelial cells but only in KO wounds (Figure 4G and Supplemental Figure S4A), confirming its regulation by miR-29. Blood vessels in the granulation tissue of KO wounds had distinct lines of punctate Lamc2–Itga6 interactions, suggesting that greater levels of Lamc2 may contribute to the adhesion of endothelial cells and, thus, improve angiogenesis (Figure 4H). Human LAMC2 and ITGA6 are confirmed targets of miR-29 in cancer cells, where LAMC2 interacts with ITGA6 to improve focal adhesion formation and cell migration.^13^ As the deletion of miR-29a/b1 resulted in the increased expression of Lamc2, this finding suggests a role for LAMC2 deposition and its interaction with ITGA6 of endothelial cells in the angiogenesis of healing wounds.

**Figure 5:**
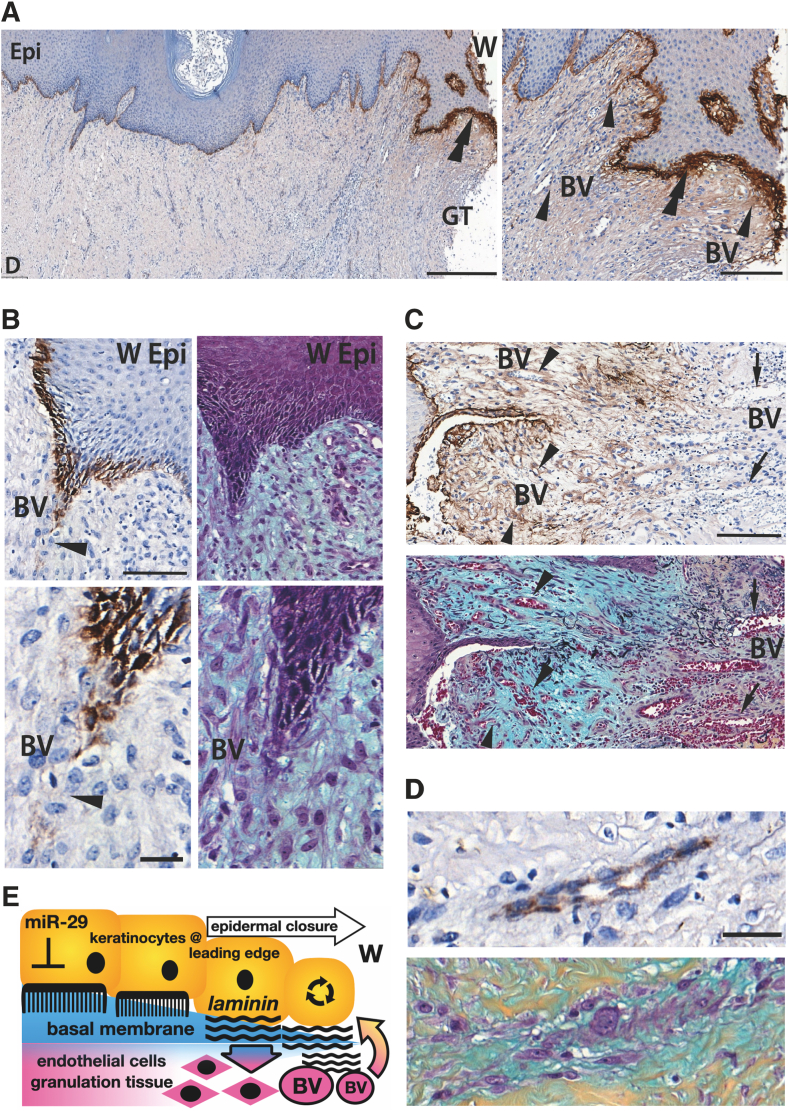
LAMC2 deposition in the matrix of human traumatic wounds. **A:** Section of a human wound biopsy specimen, stained with anti-LAMC2 antibody and counterstained with hematoxylin. Note the brown signal for LAMC2 between the epidermis (Epi) and dermis on the wound (W) edge (**double arrowheads**) and around the small blood vessels (BV, **single arrowheads**). **B:** Basal keratinocytes of the wound epidermal (W Epi) tongue deposit LAMC2 near blood vessels (BV; **single arrowheads**). **C** and **D:** Blood vessels in maturing wound matrix retain LAMC2 (**arrows**). Blue staining is immature wound matrix, which turns golden brown as more collagen is formed. Epithelial and endothelial cells appear deep purple, along with lighter purple staining of other cell types in the dermis (fibroblasts and immune cells). Erythrocytes are in red. **E:** Suggested mechanism of action of miR-29 and LAMC2 in wound repair. Deposition LAMC2 into wound bed by keratinocytes at the leading edge contributes to angiogenesis, which, in turn, feeds epidermal advancement and wound repair. Scale bars: 200 μm (**A**); 100 μm (**B**, top; **C**); 20 μm (**B**, bottom; **D**). GT, granulation tissue.

**Supplemental Figure S5:**
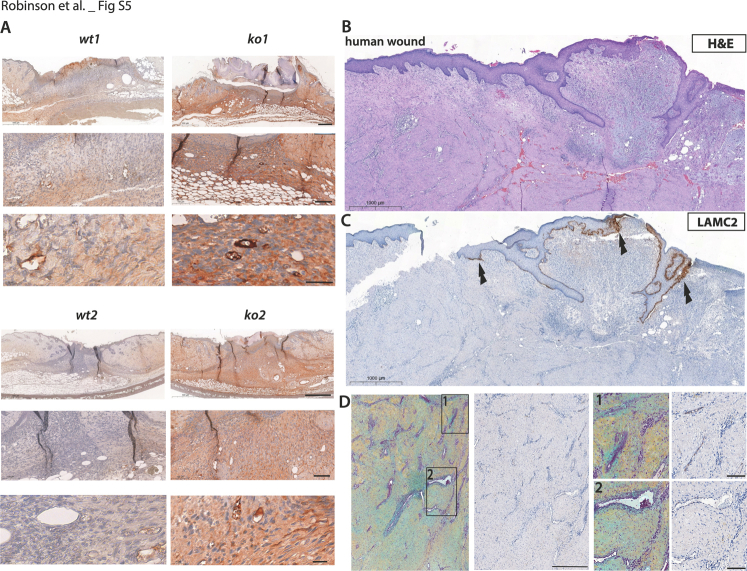
LAMC2 staining in the granulation tissue and blood vessels of wounds from WT and KO mice (**A**) and from human skin (**B**) at day 5 [stained with Lamc2 and counterstained with hematoxylin and eosin (H&E)]. **C** and **D:** Staining with LAMC2 (**C**) and pentachrome and LAMC2 (**D**) in human wounds. Note the amount of LAMC2 deposited by basal keratinocytes of the epidermis at the wound edges (**double arrowheads**). Scale bars: 500 μm (**A**, top panels; **D**, left); 100 μm (**A**, middle panels; **D**, right); 20 μm (**A**, bottom panels); 1000 μm (**B** and **C**).

To test whether this mechanism contributes to skin repair in humans, LAMC2 localization in human wounds was checked. Interestingly, greater LAMC2 was detected at the leading edge of the epidermis compared to the undisturbed epidermal–dermal interface located further from the wound (Figure 5, A and B). As expected, significant deposition of LAMC2 was found in the nascent BM forming between the epidermis and dermis at the edge of the regenerating epidermis (Figure 5, A and B). Pentachrome staining of the same area revealed signs of immature matrix, such as the formation of small blood vessels and the presence of proteoglycans (Figure 5C), with hematoxylin staining showing the presence of perfused blood vessels of various sizes immediately under the leading edge of the epidermis, and between the Rete ridges, where the lamina in small blood vessels could be interacting with the basal lamina in the epidermis (Supplemental Figures S4B and S5, B and C). As also seen in KO wounds, LAMC2 was localized around blood vessels deep in the wound granulation tissue (Supplemental Figure S5) and in some instances showed very strong staining in the blood vessel BM (Figure 5D and Supplemental Figure S5A). As the wound matrix matured (demonstrated by the golden-brown color of the fully constructed collagens in the extracellular matrix), LAMC2 remained around some blood vessels (Figure 5D) but also stained less intensely (Supplemental Figure S5D). Some parts of human wounds, however, showed a very low intensity of LAMC2 signal, resembling that in mouse WT wounds (Supplemental Figure S5, A and C). This finding suggests a transient but crucial role of LAMC2 in angiogenesis during cutaneous wound healing, which is enhanced by the inhibition of miR-29 and a subsequent increase in LAMC2.

## Discussion

These findings are important for the development of new wound therapies, especially as the addition of LAM332 has been shown to promote angiogenesis in three-dimensional co-cultures of keratinocytes and endothelial cells used for the development of biomedical implants.^31^^,^^32^ Similar to the present findings in wounds, LAMC2 was the only LAM332 subunit with increased expression in vascular endothelial cells isolated from human keloid skin, where LAMC2 expression was positively correlated with vascularization.^33^ Notably, the up-regulation of *LAMC2*, but not of other mRNAs coding for LAM332, was previously observed in a skin chemical injury model.^34^

This study was focused only on LAMC2, as it was the only laminin isoform in which expression at the mRNA and protein levels was changed in response to the inhibition of miR-29. The translational product laminin γ2 exists in two forms: heterotrimer (Lm-332γ2) and monomeric (Lm-γ2).^34^ Whether the changes in LAMC2 (encoding for Lm-γ2 chain) contribute to re-epithelization, wound healing, and angiogenesis as a monomer, or whether it also involves the other two monomers of Lm-332γ2, Lm-α3 (encoded by *LAMA3*) and Lm-β3 (encoded by *LAMB3*) chains, remains unclear. In the preliminary analysis of the data in the present study, changes in LAMA3 and LAMB3 expression were not observed. It is therefore suggested that the monomeric form of Lm-γ2 is regulated by miR-29 during wound healing; however, this concept requires further investigation. In a recent study, cancer cells released the monomeric Lm-γ2 to stimulate endothelial cell permeability, growth, and angiogenesis *in vitro* and *in vivo*,^35^ while LAM332 in the extracellular matrix produced by human keratinocytes implanted in mice was directly associated with the observed increase in peri-implant angiogenesis and implant neovascularization.^31^ It is suggested that the recombinant Lm-γ2 (or the miR-29 inhibition that increases LAMC2 in wounds), in a fashion similar to that of invading cancer cells, might stimulate invasion of the epidermal tongue into the wound bed. It would be interesting to develop an epidermis-specific miR-29 KO mouse model under keratin K14 or K16 promoter to test whether the deletion of miR-29a/b1 results in a stronger release of Lm-γ2.

This study reveals a previously unreported role of LAMC2 in wound angiogenesis, where it could stimulate the formation of blood vessels by regulating adhesion with the integrins expressed in endothelial cells. The results also suggest that LAMC2 deposition at the wound leading edge by keratinocytes enhances the formation of blood vessels, which, in turn, promotes wound re-epithelialization, forming a positive feedback loop to improve epidermal closure (Figure 5E). The findings from this model are consistent with previous findings in which high levels of LAMC2 promoted permeability of blood vessels through the interaction between LAMC2 N-terminal epidermal growth factor–like repeat domain with cell surface receptors, leading to delocalization of VE-cadherin and β-catenin from the intercellular junctions between endothelial cells.^35^ As the granulation tissue matures and the epidermal BM is re-established, the leading edge of the migrating epidermal tongue proceeds forward, depositing more LAMC2 into the immature granulation tissue. This deposition may promote further migration of keratinocytes and stimulate vascularization in the vicinity of the wound epidermis. Deposition of Lamc2 in the granulation tissue could be a provisional way to promote the formation of blood vessels before the epidermis–dermis barrier is fully re-established. This way, LAMC2 deposition contributes toward the re-establishment of the BM of epithelial and endothelial cells during the progression of the skin repair process. This presumed mechanism of angiogenesis is a faster strategy toward complete wound closure in comparison to a *de novo* synthesis and deposition of the isoform of laminin found in mature blood vessels, LAM511.^36^

Taken together, the present findings demonstrate a new role for miR-29 in epidermal repair and suggest that the release of miR-29 targets, particularly LAMC2, promotes wound healing. As miR-29 binding of the *LAMC2* 3′UTR is conserved in both mice and humans, the inhibition of miR-29 and/or overexpression of LAMC2 could thus be a new and effective strategy for improving skin repair.
